# Engaging Adolescents to Inform the Development of a Mobile Gaming App to Incentivize Physical Activity

**DOI:** 10.2196/resprot.8113

**Published:** 2017-08-29

**Authors:** Lizzy Pope, Bernice Garnett, Marguerite Dibble

**Affiliations:** ^1^ University of Vermont Burlington, VT United States; ^2^ Game Theory Burlington, VT United States

**Keywords:** adolescents, qualitative research, mHealth, physical activity

## Abstract

**Background:**

Involving youth in the development of a mobile game designed to increase physical activity may increase relevancy and adoption.

**Objective:**

To share the development process used to create a gaming app aimed at incentivizing physical activity in high school students.

**Methods:**

Five focus groups were conducted with high school students (N=50) to understand gaming behaviors. A subset of students from the focus groups chose to complete a Web-based survey (N=10). Four different versions of gaming artwork and concept design based on student input were pilot tested (N=35), and group consensus building determined the direction of the game. The 4 game versions differed in their artwork style and gaming concept with some requiring competition versus cooperation, or being more individual versus team based. Group consensus building meant that all artwork and game concept options were displayed at the front of a classroom. Students could then vote for their top artwork and concept choices by putting stickers on the top 1 or 2 artwork and concept options that they liked best. Once all votes were cast, investigators discussed the voting results with students, and brainstormed ways to incorporate popular aspects of the 3 “losing” artwork and game concepts into the winning ideas.

**Results:**

Focus group transcripts were analyzed for common themes. Artwork and gaming concept-voting data was tallied at the time of voting to share with students in real time. Focus groups and survey results revealed important themes for a successful gaming app: (1) competition, (2) balanced in-game rewards, (3) accessibility, and (4) aesthetic features. Consensus voting indicated the popularity of a collaborative competitive content design (35/66, 53%) and playful art (27/71, 38%).

**Conclusions:**

To ensure saliency and effectiveness of game-based physical activity interventions, youth need to be included in design and implementation. Furthermore, the unique preferences and social constructs of high school students need to be considered during intervention development.

## Introduction

Among American adolescents there continues to be high rates of those who are overweight and obese. This is a concern for health practitioners. According to the Centers for Disease Control and Prevention (CDC), 34.5% of youth age 12 to 19 years old were overweight or obese in 2011 to 2012 [1]. Addressing the current prevalence of overweight/obesity in adolescents is paramount as excess weight in adolescence often translates to excess weight in adulthood [2]. The positive effects of physical activity on health outcomes, learning outcomes, and social outcomes have been elucidated in a variety of previous research studies [3]. However, physical activity declines steeply from childhood to adolescence, with 27.1% of high school students nationally meeting the CDC guideline of 60 minutes of moderate/vigorous physical activity (MVPA) per day in 2013 [4]. In one recent study, adolescents only obtained an average of 39.4 minutes of MVPA per day [5].

Previous research has indicated that not having enough time and wanting to do other things are two of the most common barriers to physical activity for high school students [6]. Monetary incentives may be very effective at encouraging physical activity in both college and elementary school students as well as adults, and therefore help address the time and attention barrier to physical activity cited by adolescents [7-9]. Very few interventions have examined how to increase physical activity school wide for high school students, or ways that the behavioral theories behind incentives can be leveraged without the use of monetary incentives. Incentives inherent in a mobile game may be a feasible way to encourage high school students to be more active, and incur the positive benefits of physical activity on health, academic, and social outcomes. Using a gamification model where real-world physical activity translates into immediate “rewards” in a mobile game may successfully incentivize physical activity in high school students. Because games involve accumulating points, moving up levels, or leaderboard social recognition, they inherently provide an incentive to continue doing whatever activity results in more points, more progress, or more prowess [10]. As many high school students are familiar with and enjoy games on either their mobile devices, tablets, or gaming systems, using a gaming context to promote physical activity may be novel, age-appropriate, and widely scalable. The objective of this paper was to provide an overview of the development process used to design a gaming app that incentivized real-world physical activity with in-game rewards.

## Methods

### Recruitment and Sample

To design a physical activity gamification program for diverse adolescents; partnering with youth in the design and implementation of the program is necessary to ensure relevancy and program ownership. Youth Participatory Action Research (YPAR) is a methodology in which youth are recruited as research partners to collaboratively assess the needs/assets of their community, inform program design and implementation, as well as assist in analysis and dissemination activities [11]. To this end, YPAR served as an overarching theoretical and methodological framework for our program design and evaluation. The overall objectives of the surveys and focus groups conducted were to: (1) understand the current gaming behaviors and attitudes of the target population, (2) solicit input on barriers and facilitators of their physical activity patterns, and (3) gauge interest and feasibility in integrating physical activity into a Web-based gaming app.

Sophomore and junior students from 5 teacher advisory periods were recruited to participate in focus groups and surveys in a diverse high school in the Northeast (482/1006, 47.91% free/reduced lunch; 362/1006, 35.98% non-White; 181/1006, 17.99% English language learners). A letter was sent home to parents where they could opt out of having their student participate in the study. Students also provided verbal consent to participate in focus groups, and had the option to decline to participate. The University of Vermont’s Committee on Human Research in the Behavioral and Social Sciences approved the study. A total of 5 focus groups were conducted with sophomore and junior students during the fall of 2015 (N=50) to understand gaming behaviors and preferences. School staff and researchers decided on 5 focus groups, as it was thought that 5 groups would allow for ample exposure to students’ varying ideas and perspectives without disrupting the normal school schedule. Focus groups were conducted during the 30-minute teacher advisory period that sophomores and juniors attend daily. The focus groups were led by one of the researchers and the game designer, while a second researcher took notes and recorded responses. Each focus group followed the same script of questions, while also allowing follow-up on new ideas raised by each particular group. The focus groups averaged 10 students each. At the conclusion of the focus groups, all participating students were emailed a 10-question survey that contained similar questions to the focus groups. This survey addressed the types of games and physical activity that students engaged in at school and at home, and was developed specifically for the study. It was thought that students who may have been too shy to speak up during the focus groups could use the survey as a chance to share their thoughts. A subset of 10 students filled out the parallel qualitative survey.

**Figure 1:**
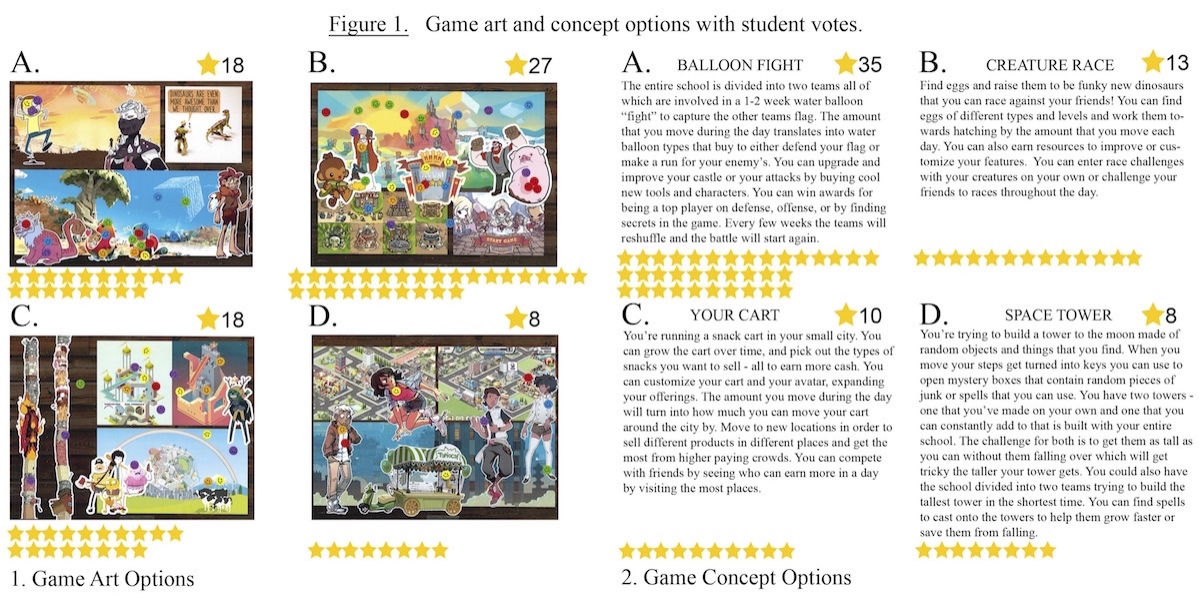
Game art and concept options with student votes.

After the initial focus groups, the research and game design team incorporated student input, theory, and best practices from the gaming and behavioral economics fields to design 4 game content schemas and 4 artwork styles for the students to vote on separately using consensus voting techniques. During the spring of 2016, a total of 35 students from the original 5 teacher advisory periods were assembled to vote on artwork and content gaming schema. Each student was given 4 colored stickers, 2 to place on their favorite artwork panels and 2 to place on their favorite content gaming schemas [12]. Both the artwork and content gaming schema had 4 different options for the students to choose from (see Figure 1).

Game concepts were developed based on previous research on game design and behavioral economics. Each concept was designed to capitalize on principles in basic psychology known to increase motivation, including reinforcement, challenge, cooperation, competition, identity, and surprise [13]. The game concepts were also designed to provide intermittent reinforcement, an incentive strategy known to help guard against behavior extinction [9,14]. Finally, the game concepts were designed to leverage behavioral economic theory by appealing to decision-making heuristics like loss aversion (the tendency to avoid experiencing losses) and the endowment effect (the higher value assessment of things you own vs things you do not own) that humans subconsciously use frequently [10]. Although all 4 game concepts emphasized various gaming engagement techniques, they each incorporated a variety of motivational elements, and differed mostly in the scenario and mechanics proposed. At the conclusion of the voting, researchers discussed the results with the students, and gathered information on elements of each style that students liked.

### Analysis

Focus groups were audio recorded and transcribed. Based on theory, previous research, the focus group protocol, and the use of key words in context (eg, the use of in vivo coding) a codebook was developed to identify salient themes. Transcripts were analyzed using this codebook. We followed a coding schema developed by Saldaña [15] in which first cycle codes, including descriptive codes that were used to identify the basic topic of a passage as well as process codes that refer to conceptual action in the data, such as game aesthetics, were applied followed by second cycle codes, or pattern codes, to understand relationships among codes. Transcriptions were coded based on thematic analysis to uncover consistent and major trends as they related to gaming and physical activity attitudes and behavior [16]. Results from the 10 qualitative survey responses were analyzed using the codebook generated from the focus group analysis. Voting was tallied across the artwork and gaming content to determine the majority vote.

## Results

### Qualitative Results

Focus groups and survey results with youth revealed several themes important for game design: (1) competition, (2) balanced in-game rewards, (3) accessibility, and (4) aesthetic features. Textbox 1 illustrates the major themes from the focus group and survey results aligned with salient quotes to further illustrate youth voice.

Youth described the importance of having an element of competition, particularly a competition with friends or within the school community incorporated into the game design. Additionally, to ensure fairness within the game, youth described various game design features to ensure “balanced in-game rewards” to motivate and reward students who were not as physically active as others. Finally, youth described the importance of accessibility, both technologic and content accessibility, to encourage multiple segments of their demographic to play and continue playing a game. Students also mentioned the importance of “cool graphics” and other aesthetics, specifically cited as critical when trying to encourage someone to play a new game.

### Consensus Voting

Consensus voting of students indicated the popularity of a collaborative competitive content design was 53% (35/66) and playful art was 38% (27/71), compared with the other content and art design options presented.

**Results and salient quotes from the focus groups related to gaming content and design.**

Focus group themes: game design

1. Competitions:
  - “Like a school wide capture the flag...”
  - “If you can create a game that somehow tracks your steps or activity and then you can compete with that and it can motivate”
2. Balanced in-game rewards:
  - “Progress to be a part of a competition so people don’t get discouraged”
  - “There are a lot of games where points can earn you special cash where you can buy special things with and like if you run more you could get special cash”
3. Accessibility:
  - “Any free app that you can get access to that was simple and obnoxious...”
  - “Compatibility with a lot of difference devices”
  - “You have to have something that is really accessible to all, like you could have someone that has been sedentary their entire life and then you could have someone like the captain of the varsity track team and they are in the same school and competing in the same game you are making so you have to have something that...it should probably be targeted to kids who don’t do anything...rewarding progress”
4. Aesthetic features:
  - “If it has a nice looking icon and the name sounds like a real app and not some that is fake or suspicious”
  - “I always think it’s really cool when you can customize because like there are games that you do what they design and there are games that you can customize to how you like it which is pretty awesome”

## Discussion

### Importance of Youth Engagement in Intervention Design

To ensure that mHealth and eHealth technologies are effective and resonate with the target population, efforts to incorporate the perspectives, attitudes, and ideas of the target demographic should be solicited and embedded within mobile technologies to improve health. Explicitly incorporating youth voice in the design and construction of eHealth and mHealth interventions is a nascent applied research area [17,18]. Although there are many health and fitness apps available, very few capitalize on effective gaming strategies and behavioral theory [19]. The development process of the current game was designed to use established mixed-methods research, and determine which gaming and health promotion elements were most appealing to an adolescent population, an area of research that has not been widely explored previously. The successful artwork and game concept themes identified are familiar to game designers [19], but not commonly incorporated into physical activity promotion interventions for adolescents, which are themselves sparse and largely consist of curriculum development [20]. Our formative research indicates that gamification is of great interest to adolescents and could be used to add the important element of “fun” to behavior-change interventions. If an intervention is more pleasurable to engage with, it is much more likely that it can be effective in the long-term.

Engaging our target demographic population resulted in important directions for game design, artwork, and integration of physical activity into the game interface. Youth described the importance of aesthetics and accessibility that would encourage them to begin playing a new game, and then provided concrete examples of game design features that would keep them actively engaged and motivated in a physical activity–integrated gamification interface. The 4 themes that emerged from our focus group work helped drive intervention development in ways that were both predictable and more unexpected. For example, the research team was not surprised that competition, rewards, accessibility, and aesthetic features were the predominant themes to emerge from the focus groups. However, the specific manifestations of certain thematic elements suggested by the target audience were often innovative or surprising. The research team believed that an edgy, abstract style was the most attractive artwork style, yet youth overwhelmingly voted for the playful artwork style, illustrating the importance of gathering youth feedback on all aspects of intervention development. Furthermore, youth really wanted to be rewarded for not just goal achievement, but improvement and progress toward goal achievement. Research in business and health has indicated that rewarding progress is a very effective way to keep people on track with their goals, as small rewards for improvement or progress prevent discouragement and motivate continued behavior completion [21,22]. Therefore, it was very interesting that the adolescents could identify progress-based incentives as an important aspect of any game.

In addition to wanting to be rewarded for progress, multiple students also suggested a “capture the flag” style game play design, something that the research team may not have given much thought to without youth involvement. The interest in integrating team-based competition into a game concept may reflect the dominant nature of the social-emotional reward style in adolescents. More so than adults or children, neuroscience research indicates that adolescents’ neural reward system responds to social stimuli [23,24]. The social nature of a team-based competitive game where adolescents collaborate with friends or classmates to work toward a common goal may be particularly appealing. It has also been shown that during adolescence there is a shift from self-oriented behavior to more prosocial behavior, which further illuminates why a competitive team-based gaming concept would be popular [24]. This shift to more prosocial decision-making and ability to take the perspective of another person may partly explain why accessibility and fairness were important gaming concepts for focus group participants. Adolescents wanted peers of all fitness levels to be able to succeed in the game and they also wanted rewards for progress, indicating the importance of allowing even nonathletes to succeed in a physical activity–oriented game. Additionally, adolescents are known to be risk takers with their decision-making [23-25]. This preference for risk may result in wanting to play a game where you’re part of a team and there is social pressure to perform a certain behavior so that your team wins.

The mixed-methods structure of the proposed intervention is novel and timely. Mixed-methods research helps provide a more tailored and efficacious intervention, as well as a more complete picture of how intervention outcomes arise. The YPAR method gives the intervention the best chance of success by involving future participants in the development of an engaging, relevant, and appropriate intervention. The YPAR method also allowed us to cultivate intervention champions organically. By involving students throughout the game development year, we began the process of building excitement for the gaming intervention and buy-in to the app.

### Strengths and Limitations

This study had several strengths. First, this study included gathering feedback from a diverse group of adolescents at a public high school. Secondly, the multiple modalities used to collect input and feedback also greatly enhanced the study by allowing students to express their opinions in a variety of ways. Finally, the ability of our game design team to talk gaming specifics with the adolescents, and our research team to systematically collect qualitative data both increased the usefulness and rigor of our results. Limitations of the study include the rather small sample size, and the inclusion of only sophomores and juniors. Future work could examine differences in using YPAR methodology in larger samples and age ranges.

## Abbreviations

CDC: Centers for Disease Control and Prevention
MVPA: moderate/vigorous physical activity
YPAR: youth participatory action research

## References

[1] Ogden CL, Carroll MD, Kit BK, Flegal KM (2014). Prevalence of childhood and adult obesity in the United States, 2011-2012. JAMA.

[2] Guo S, Roche A, Chumlea W, Gardner J, Siervogel R (1994). The predictive value of childhood body mass index values for overweight at age 35 y. Am J Clin Nutr.

[3] Janssen I, Leblanc AG (2010). Systematic review of the health benefits of physical activity and fitness in school-aged children and youth. Int J Behav Nutr Phys Act.

[4] Kann L, Kinchen S, Shanklin SL, Flint KH, Kawkins J, Harris WA, Lowry R, Olsen EO, McManus T, Chyen D, Whittle L, Taylor E, Demissie Z, Brener N, Thornton J, Moore J, Zaza S, Centers for Disease ControlPrevention (CDC) (2014). Youth risk behavior surveillance--United States, 2013. MMWR Suppl.

[5] Carlson JA, Schipperijn J, Kerr J, Saelens BE, Natarajan L, Frank LD, Glanz K, Conway TL, Chapman JE, Cain KL, Sallis JF (2016). Locations of physical activity as assessed by GPS in young adolescents. Pediatrics.

[6] Tergerson J, King K (2002). Do perceived cues, benefits, and barriers to physical activity differ between male and female adolescents? Journal of school health. J Sch Health.

[7] Pope L, Harvey J (2014). The efficacy of incentives to motivate continued fitness-center attendance in college first-year students: a randomized controlled trial. J Am Coll Health.

[8] Garnett B, Becker K, Vierling D, Gleason C, DiCenzo D, Mongeon L (2016). A mixed-methods evaluation of the before-school incentive-based physical activity programme. Health Educ J.

[9] Jeffery R (2013). Financial incentives and weight control. Prev Med.

[10] King D, Greaves F, Exeter C, Darzi A (2013). 'Gamification': influencing health behaviours with games. J R Soc Med.

[11] Ozer E, Douglas L (2013). The impact of participatory research on urban teens: an experimental evaluation. Am J Community Psychol.

[12] McMurray A (1994). Three decision-making aids: brainstorming, nominal group, and Delphi technique. J Nurs Staff Dev.

[13] Dibble M (2014). TEDx.

[14] Skinner B (1953). Science and Human Behavior.

[15] Saldaña J (2013). The Coding Manual for Qualitative Researchers.

[16] Boyatzis R (1998). Transforming Qualitative Information: Thematic Analysis and Code Development.

[17] Hingle M, Nichter M, Medeiros M, Grace S (2013). Texting for health: the use of participatory methods to develop healthy lifestyle messages for teens. J Nutr Educ Behav.

[18] Mojica CM, Parra-Medina D, Yin Z, Akopian D, Esparza LA (2014). Assessing media access and use among Latina adolescents to inform development of a physical activity promotion intervention incorporating text messaging. Health Promot Pract.

[19] Lister C, West JH, Cannon B, Sax T, Brodegard D (2014). Just a fad? Gamification in health and fitness apps. JMIR Serious Games.

[20] Dobbins M, DeCorby K, Robeson P, Husson H, Tirilis D (2009). School-based physical activity programs for promoting physical activity and fitness in children and adolescents aged 6-18. Cochrane Database Syst Rev.

[21] Amabile T, Kramer S (2011). The power of small wins. Harvard Business Review.

[22] Terry P (2012). The advantage of progress-based incentives. Managed Care.

[23] Galvan A (2010). Adolescent development of the reward system. Front Hum Neurosci.

[24] Crone EA, Dahl RE (2012). Understanding adolescence as a period of social-affective engagement and goal flexibility. Nat Rev Neurosci.

[25] Smith AR, Chein J, Steinberg L (2013). Impact of socio-emotional context, brain development, and pubertal maturation on adolescent risk-taking. Horm Behav.

